# Prognostic value of FGFR1 expression and amplification in patients with HNSCC: A systematic review and meta-analysis

**DOI:** 10.1371/journal.pone.0251202

**Published:** 2021-05-14

**Authors:** Yao Hu, Li-Sha Ai, Liu-Qing Zhou

**Affiliations:** 1 Department of Otorhinolaryngology, The Central Hospital of Wuhan, Wuhan, China; 2 Department of Hematology, Union Hospital, Tongji Medical College, Huazhong University of Science and Technology, Wuhan, China; 3 Department of Otorhinolaryngology, Union Hospital, Tongji Medical College, Huazhong University of Science and Technology, Wuhan, China; Roswell Park Cancer Institute, UNITED STATES

## Abstract

Fibroblast growth factor receptor 1 (FGFR1) has recently been identified as a promising novel therapeutic target and prognostic marker in different types of cancer. In the present study, a meta-analysis was performed to clarify the correlation between FGFR1 and the survival outcomes of head and neck squamous cell carcinoma (HNSCC) patients. PubMed, Embase, and Web of Science were systematically searched for relevant studies in order to explore the prognostic significance of FGFR1 in HNSCC. Hazards ratios (HR) and 95% confidence intervals (CI) were collected to estimate the correlation between overexpression and amplification of FGFR1 and survival outcomes of HNSCC patients. Nine studies including 2708 patients with HNSCC were finally selected for the meta-analysis. The results indicated that FGFR1 predicted poor overall survival (OS) (HR, 1.97; 95% CI, 1.49–2.61, P<0.001) in HNSCC patients. Futhermore, FGFR1 was related to poor OS in human papillomavirus (HPV) negative HNSCC not in HPV positive HNSCC patients. Subgroup analysis stratified by molecular abnormalities, such as overexpression or amplification showed the similar results. The present study demonstrated that HNSCC patients with FGFR1 overexpression and amplification were more likely to exhibit poorer survival.

## Introduction

Head and neck squamous cell carcinoma (HNSCC) develop from the mucosal linings of the upper aerodigestive tract, comprising 1) the nasal cavity and paranasal sinuses, 2) the nasopharynx, 3) the hypopharynx, larynx, and trachea, and 4) the oral cavity and oropharynx. Squamous cell carcinoma (SCC) is the most frequent malignant tumor of the head and neck region. Although the group of malignancies arise from different sites of head and neck region, they have similar pathogenesis, staging system, therapeutic strategy, and prognosis. Therefore, it is rational to classify them into one category, HNSCC [1]. HNSCC accounts for approximately 3% of new cancer cases annually and is the fifth most common cancer in the world [2,3]. Despite the successful development of treatment methods, such as surgery, chemotherapy, immunotherapy and radiation, the five-year survival rate of HNSCC patients is estimated to be between 40 and 50% [4]. SCC accounts for more than 95% of these cancers, while their molecular and clinical characteristics are heterogeneous [4–6]. The lack of significant improvement in the survival rates of HNSCC patients with the current treatment methods has led to a search for new prognostic biomarkers and therapeutic targets [7,8].

The fibroblast growth factor receptor (FGFR) family comprises four main receptors (FGFR1-4), which are structurally associated with the corresponding receptor tyrosine kinases. FGFRs are involved in several cellular processes including angiogenesis, wound healing, tissue repair and tumorigenesis [9,10]. Substantial evidence further indicates that genomic driver aberrations, such as mutations, amplifications and translocations may cause a dysregulation in the FGF-FGFR pathway and play an important role in tumor development [11]. Molecular abnormalities and overexpression of FGFR1 have been associated with poor outcomes and have been described in lung carcinomas [12], breast cancer [13], pancreatic cancer [14], oral squamous carcinoma [15] and esophageal squamous cell carcinomas [16]. Moreover, human papillomavirus (HPV) status is known to be associated with both FGFR1 expression/amplification and survival outcomes in HNSCC [17,18].

However, the direct impact of FGFR1 expression and amplification on HNSCC patient survival remains inconclusive due to the variance in the sample size and the experimental design of the studies performed. In the present study, the databases of PubMed, Embase and Web of Science were searched for relevant publications and a meta-analysis was performed. The objective was to assess the association between FGFR1 and the survival outcomes in HNSCC patients.

## Materials and methods

### Search strategy

We searched for articles published between 2000 and 2020. The complete literature search was conducted in June 2020 in accordance with Dickersin *et al* [19]. The search was performed throughout specific databases, including PubMed, Web of Science and EMBASE. The search terms included the following: (FGFR1 or fibroblast growth factor receptor 1) and (prognosis OR outcome OR recurrence OR survival OR mortality OR progression) and (head and neck or oral or laryngeal or tonsil or oropharyngeal or oropharynx) and (cancer or squamous cell carcinoma). Furthermore, the reference lists of the retrieved studies were evaluated manually to identify potential pertinent publications.

### Selection criteria

The inclusion criteria of the meta-analysis were the following: 1) patients diagnosed with HNSCC; 2) association between the overexpression and amplification of FGFR1 and overall survival (OS); and 3) the language of publications that was confined to English. 4) Sufficient statistical analysis was required, including hazard ratios (HR) and the 95% confidence interval (CI) and HR with prognostic endpoints, or data that could be used to estimate the HR and 95% CI, or other outcomes for OS such as Kaplan- Meier survival curves. The exclusion criteria were the following: 1) studies without sufficient data for meta-analysis; 2) abstracts, reviews, letters and case reports; and 3) studies with non-specific data regarding HNSCC or FGFR1. In case the same cohort was reported by several publications, the most recent publication was included in the meta-analysis.

### Data extraction

First, we inspected the repeatability, and removed the repeated papers. Then, titles and abstracts of the papers were perused carefully. Finally, reading all enrolled full articles carefully again to confirm the exactly appropriate studies. Two investigators independently evaluated the literature against the inclusion and exclusion criteria (LQ Zhou and Y Hu). Discordance in assessments were resolved by discussion with a third investigator (LS Ai). The authors of the studies were contacted by e-mail to request additional information or data for meta-analytic calculations. The Newcastle-Ottawa Scale (NOS) [20,21] was used to assess the qualities of the included publications, which uses a star system (maximum is nine stars) to evaluate a study in the three following domains: selection of participants, comparability of study groups and the ascertainment of outcomes of interest. Scores of NOS of ≥6 were considered as high-quality studies. A detailed description about how we scored each study was shown in S1 Table. The Recommendations for Tumor Marker Prognostic Studies (REMARK) was also used to access the qualities of the included studies [22]. The REMARK checklist consists of 20 items to report for published tumor marker prognostic studies.

The following information was extracted from the studies: author, year, country, cancer type, sample size, age, follow-up, method, cox proportional hazards model, survival analysis, HR, NOS score, REMARK score and OS (OS was detected from the medical treatment until the last follow-up or the time of death of the patient).

### Statistical analysis

The HR and the 95% CI were obtained directly from the primary publications or estimated by *p* values and other published data following Parmer’s methods [23]. Statistical heterogeneity among the included studies was analyzed by the χ^2^-based Q test and the I^2^ test [24]. The radom-effect model was applied in our meta—analysis, however, the estimation of between-study variance might not be precise [25,26]. Moreover, subgroup analysis was conducted to explore the source of heterogeneity. Sensitivity analysis was also performed to investigate the influence of each individual study on the overall pooled results. The Begg’s and Egger’s tests were used to evaluate the publication bias. All statistical analyses were performed with STATA statistical software version 12.0 (StataCorp Lp).

## Results

### Selection and characteristics of the included studies

**Fig 1:**
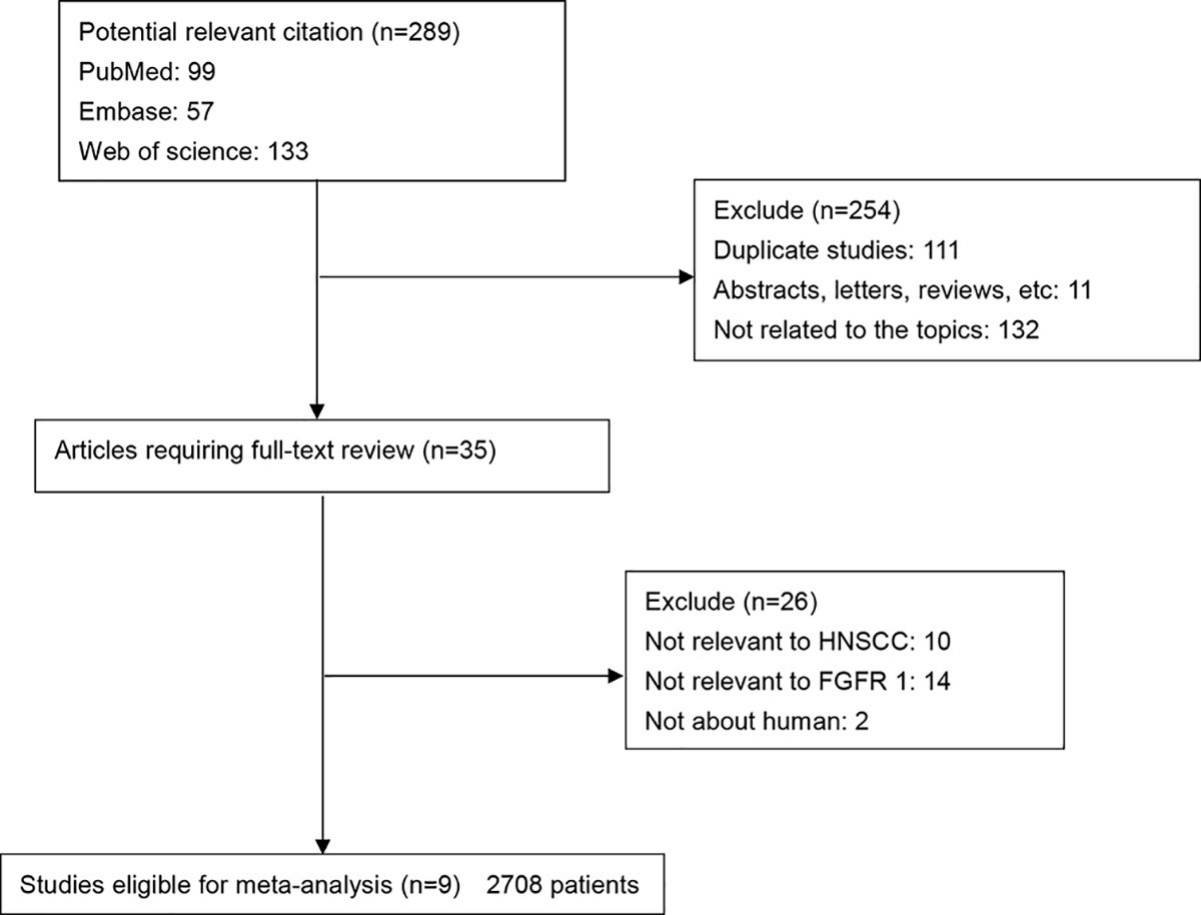
Flow diagram of the selection of relevant studies included in the meta-analysis.

A total of 289 potential records were initially identified by searching the electronic databases (Fig 1). Following exclusion of the duplicates (n = 111), reviews, abstracts and letters (n = 11) and the studies not related to the topics (n = 132), the remaining studies (n = 35) were further evaluated by reading their full texts. A total of 26 studies did not provide specific data regarding HNSCC or FGFR1 and therefore were excluded. Finally, nine studies between 2013 and 2020 with a total 2708 HNSCC patients were included in the present meta-analysis.

The characteristics of the included studies were summarized (Table 1), such as author, year, country, cancer type, sample size, age, follow-up, method, HPV infection, cox proportional hazards model, survival analysis, HR, NOS score and REMARK score. These studies were from France, Netherlands, USA, Australia, Brazil, South Korea and Poland, including three for laryngeal squamous cell carcinoma (LSCC), two for oral tongue squamous cell carcinomas (OTSCC), three for head and neck squamous cell carcinoma (HNSCC), one for oropharyngeal squamous cell carcinoma (OPSCC) and one for oral cavity squamous cell carcinoma (OCSCC), one for hypopharyngeal squamous cell carcinoma (HPSCC). Seven publications included >100 patients and two publications enrolled <100 patients. Nine studies including a total of 2708 patients reported OS. The HR and 95% CI were directly reported in eight studies and were estimated in one study in the original literature. The NOS score was >6 in eight studies. The REMARK score is between 9–16.

**Table 1:** Characteristics of the studies examined in the meta-analysis.

Author	Year	Country	Cancer type	Sample size	Age	Follow-up(months)	Method	HPV infection	Cox proportional hazards model	Survival analysis	HR	NOS score	REMARK score
Dubot [18]	2018	FRANCE	HNSCC	122	56(22–78)	60	Targeted NGS	Yes	Multivariate	OS	Reported	7	15
Koole 1 [17]	2016	Netherlands	HNSCC	452	56(35–80)	68	IHC	Yes	Multivariate	OS,DFS	Reported	8	16
Monico [27]	2018	USA	LSCC	74	NR	180	ddPCR	NR	Multivariate	OS	Reported	6	12
Young [33]	2013	Australia	OTSCC	123	59(21–93)	61.2	FISH	NR	NO	OS,PFS	Reported	5	9
Koole 2 [32]	2016	Netherlands	OCSCC	512	62(24–94)	78.5	IHC	Yes	Multivariate	OS	Reported	6	16
Koole 3 [32]	2016	Netherlands	OPSCC	439	58(35–59)	57	IHC	Yes	Multivariate	OS	Reported	6	16
Mariz [28]	2019	Brazil	OTSCC	85	55.5(19–89)	NR	IHC	NR	Multivariate	OS,DFS	Reported	6	11
Starska [29]	2018	Poland	LSCC	137	61.9(45–83)	NR	Western blotting	NR	Multivariate	OS,DFS	Reported	6	12
Kim 1 [34]	2020	South Korea	LSCC	155	64(30–88)	38.8	FISH,IHC	NR	Univariate	OS,DFS	Reported	7	12
Kim 2 [34]	2020	South Korea	HPSCC	54	64(30–88)	38.8	FISH,IHC	NR	Univariate	OS,DFS	Reported	7	12
Goke [30]	2013	USA	HNSCC	555	62	NR	FISH	YES	NO	OS	Estimated	6	10

NR, not reported; NOS, Newcastle-Ottawa Quality Scale; REMARK, Recommendations for Tumor Marker Prognostic Studies; HPV, human papillomavirus; Targeted NGS, targeted next generation sequencing; IHC, immunohistochemistry; ddPCR, droplet digital PCR; FISH, fluorescence in situ hybridization.

### Association between FGFR1 and survival of HNSCC patients

**Fig 2:**
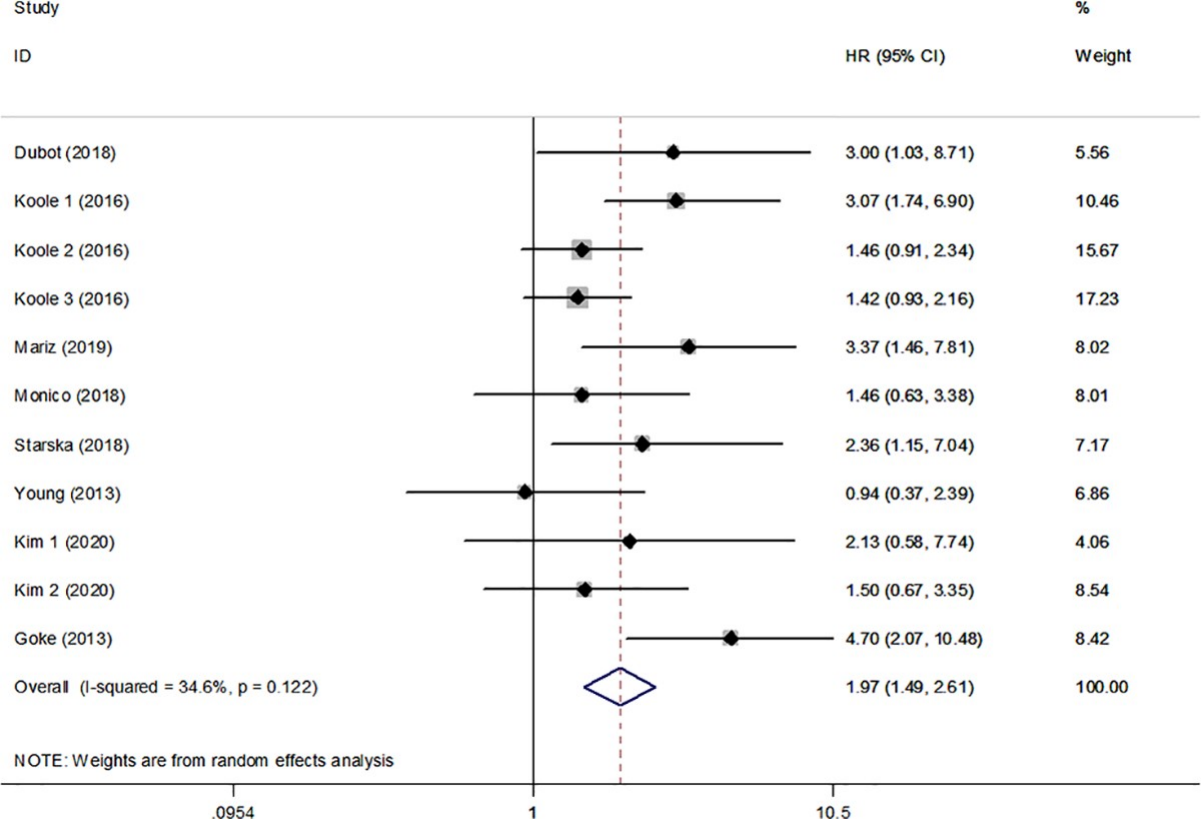
Forest plot indicating the association between overexpression/amplification of FGFR1 and OS in HNSCC. FGFR1, fibroblast growth factor receptor 1; OS, overall survival; HNSCC, head and neck squamous cell carcinoma.

Nine studies in the present analysis examined the association between FGFR1 and the survival of patients with HNSCC. The combined results of these studies indicated overexpression and amplification of FGFR1 were associated with poor OS (HR, 1.97; 95% CI, 1.49–2.61, P<0.001). Low heterogeneity was noted (I^2^ = 34.6%, P_heterogeneity_ = 0.122) (Fig 2).

**Fig 3:**
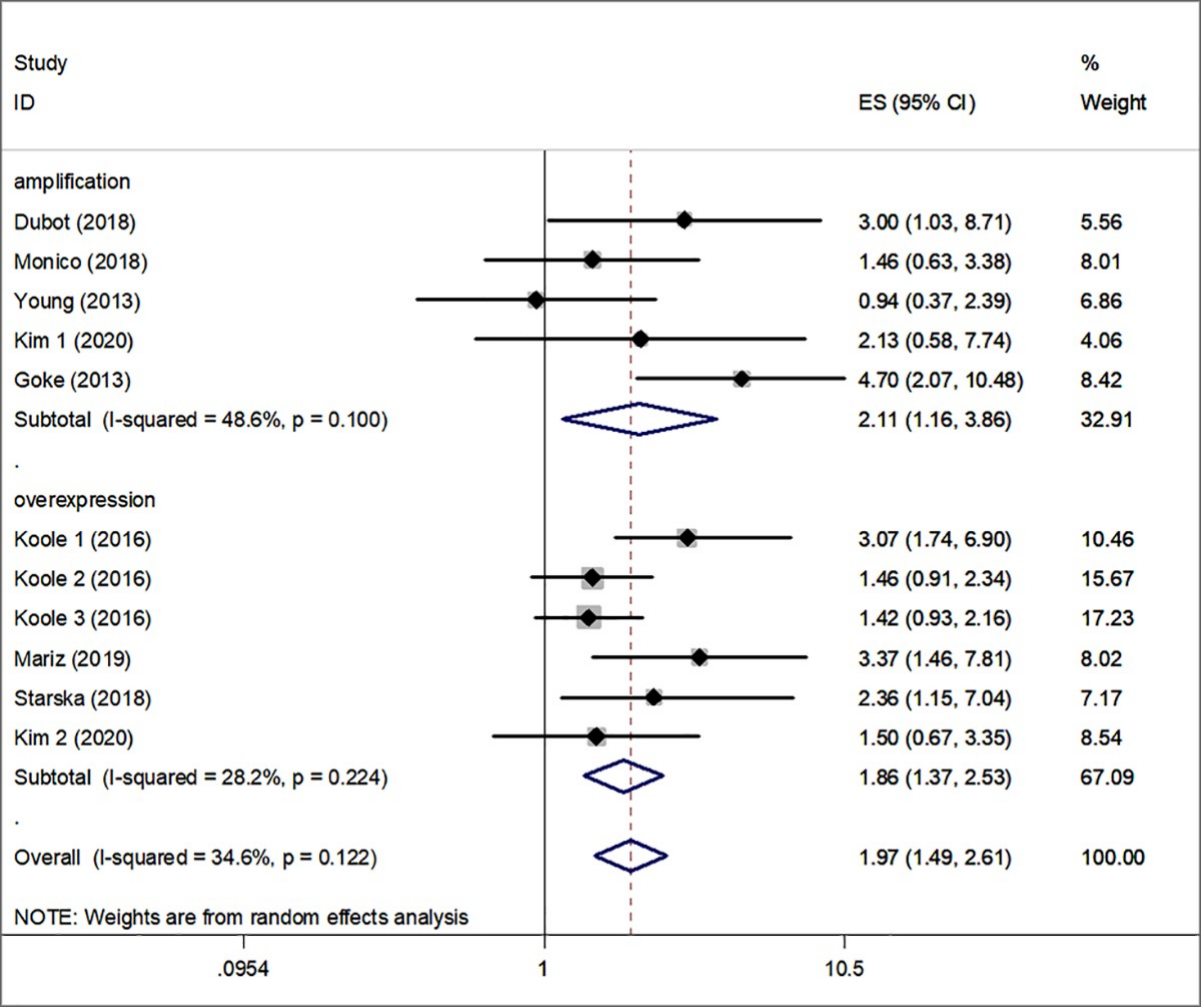
Subgroup analysis for OS was performed following stratification by FGFR1 overexpression and amplification. FGFR1, fibroblast growth factor receptor 1; OS, overall survival.

**Fig 4:**
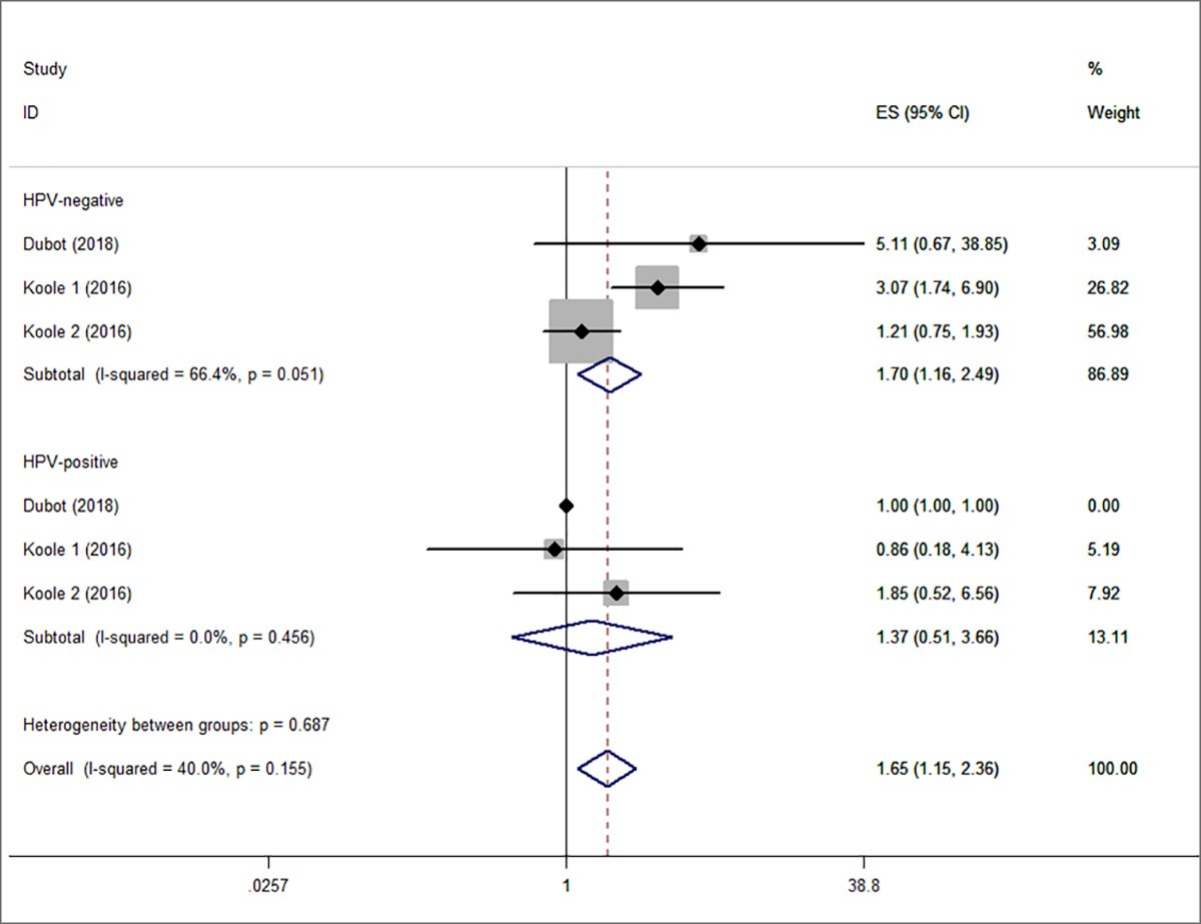
Subgroup analysis for OS was performed following stratification by HPV infection. OS, overall survival; HPV, human papillomavirus.

Subgroup analysis for OS was also performed and was stratified according to FGFR1 overexpression and amplification. The summarized HR for FGFR1 overexpression was 1.86 (95% CI, 1.37–2.53, P<0.001) and for the FGFR1 amplification 2.11 (95% CI, 1.16–3.86, P<0.001) (Fig 3). Low heterogeneity was noted between the FGFR1 overexpression and OS, medium heterogeneity was noted between the FGFR1 amplification and OS. (I^2^ = 28.2%, P_heterogeneity_ = 0.224; I^2^ = 48.6%, P_heterogeneity_ = 0.100, respectively). Furthermore, FGFR1 was related to poorer OS in HPV negative HNSCC (HR, 1.70; 95% CI, 1.16–2.49, P<0.05) (Fig 4).

### Sensitivity analysis

**Fig 5:**
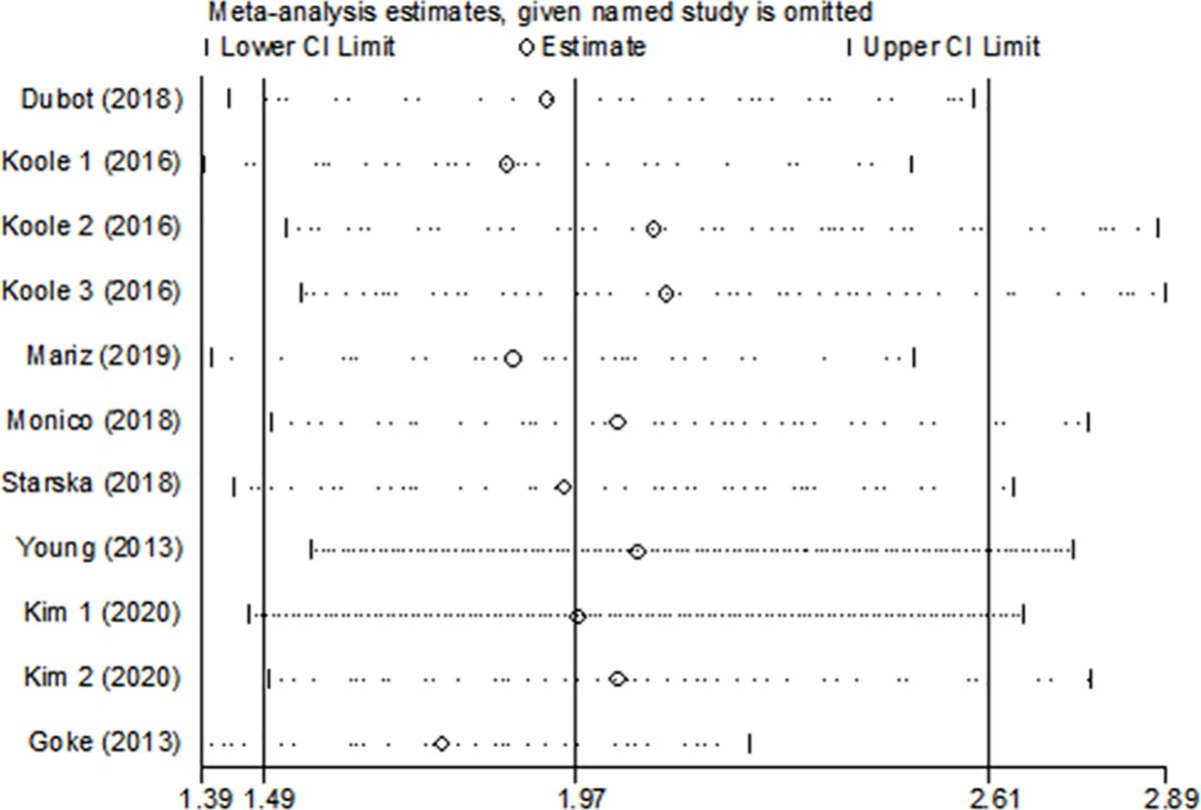
The sensitivity analyses were conducted to evaluate the effects of each single study on the overall effect.

The sensitivity analyses were conducted to evaluate the effects of each single study on the overall effect. The analysis did not detect a study that could alter significantly the combined results (Fig 5). The results of the sensitivity indicated that the pooled effect size of the meta-analysis results was stable and reliable.

### Publication bias

**Fig 6:**
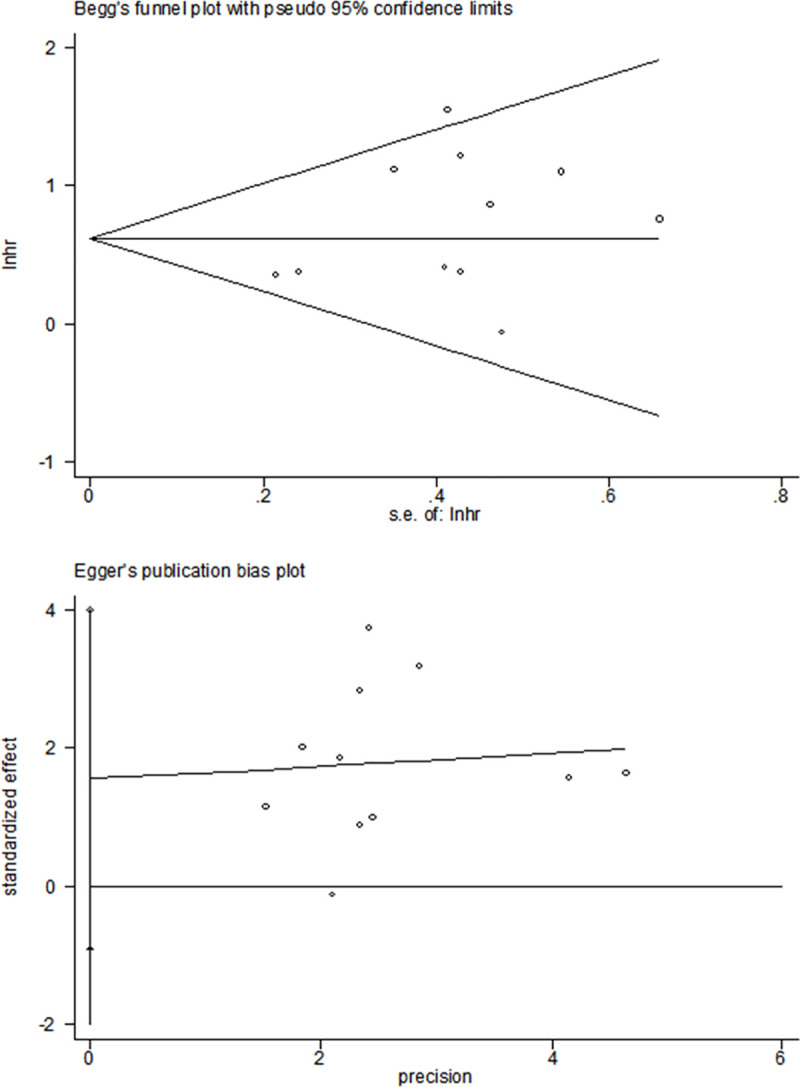
Publication bias of the enrolled analysis. The publication bias was accessed using the Begg’s funnel plots and the Egger’s test.

The publication bias was assessed by the Begg’s funnel plots and the Egger’s test in the present study. The results were quite symmetric, indicating that the analysis did not include publication bias among the studies (P = 0.187) (Fig 6).

## Discussion

Numerous studies have focused on the identification of new prognostic markers that can be used for cancer monitoring and detection. An association between FGFR1 and poor prognosis has been shown in various types of HNSCC [27–29]. Goke *et al* reported that FGFR1 amplification was a frequent event in primary and metastatic HNSCC and that it could be used as a poor prognostic indicator [30]. Dubot *et al* demonstrated that FGFR1 amplification was associated with lower survival and that it could be used as a prognostic biomarker for patients with HNSCC [18]. Hase *et al*, Young *et al* and Koole *et al* reported that FGFR1 was associated with poor outcome of HNSCC [17,31–33]. Kim *et al* recently found that FGFR1 amplification may serve as an independent prognostic factor for DFS in hypopharyngeal and laryngeal squamous cell carcinoma [34]. Our results are consistent with these results. However, Ipenburg et al reported FGFR1 gene amplification and FGFR1 protein expression are not of value as prognostic biomarkers in HNSCC in a review, [35]. The results are contrast with ours, but the evidence on FGFR1 in HNSCC is limited to only very few studies in Ipenburg’s study. These results are not comparable, due to the patient population and heterogeneous designs. The present study is the first meta-analysis including nine published studies with 2708 patients to provide useful information for clinical decision-making in HNSCC. The results indicated that overexpression and amplification of FGFR1 significantly predicted poor OS in HPV negative HNSCC patients.

A recent study reported that 7.1% of all tumor types exhibited genetic alterations and that FGFR1 was involved in almost 50% of these alterations [36,37]. A significant association between disease prognosis and FGFR1 expression was noted in various cancer patients and the studies suggested that FGFR1 was an independent prognostic factor [13,38]. The exact mechanism underlying the association of FGFR1 with cancer incidence has not been clearly elucidated. Recently, numerous studies have shown that FGFR1 can promote tumour progression and development by regulating signaling pathways and inducing cancer cell survival, proliferation and tumour angiogenesis [39,40]. Therefore, FGFR1 overexpression may be used as a marker of tumorigenesis and inflammation and as a poor prognostic indicator of cancer patients.

Targeted therapies have produced striking benefits for patients with cancer. FGFRs are not constitutively active in nonmalignant cells due to their transmembrane proteins that contain intrinsic enzymatic activities. Therefore, FGFRs are preferable targets of other therapeutic modalities and small-molecule inhibitors, such as ligand traps and antibody-based agents can inhibit FGFR signaling with therapeutic efficacy in cancer patients [41,42]. Certain types of cancers, such as endometrial uterine cancer, breast cancer [13], urothelial carcinoma [43] and lung cancer [44] have been shown to be responsive to FGFR-mediated tumour therapy. Clinical trials have provided proof that FGFR kinase inhibitors are effective therapeutic agents used in specific cancer types. According to the results of the present study, the FGFR inhibitors may hold a therapeutic potential against HNSCC.

However, the present meta-analysis contains several limitations. First, the numbers of articles used for assessing the association between FGFR1 and the prognosis of HNSCC were limited in the present meta-analysis. Therefore, additional studies are required to produce accurate conclusions. In addition, according to Begg’s funnel plots and the Egger’s test, the publication bias was not significant. Second, studies containing languages other than English and the inclusion of unpublished data may contribute to additional bias. Third, the results may be heterogeneous due to the utilization of different methods and statistical analysis. Forth, our results may be an overestimate of the prognostic significance of FGFR1 to some extent due to the majority of the included studies reporting positive results. Finally, one of the records included in the systematic review did not report the HR directly. Therefore, the extrapolated HR might be less reliable compared with reported statistics.

In summary, the present meta-analysis included electronic databases and a total of 2708 patients from nine studies. The results demonstrated that patients with overexpression and amplification of FGFR1 were more likely to exhibit poor prognosis. Taken together, the meta-analysis results suggest that FGFR1 possesses a prognostic value for HNSCC. However, additional studies with larger sample sizes are required to acquire more representative and precise findings.

## Supporting information

S1 Checklist The checklist of the NOS including selection of participants, comparability of study groups and the ascertainment of outcomes of interest. (DOC)

S1 Table Quality assessment based on the Newcastle-Ottawa Scale (NOS). (DOCX)

S2 Table The covariates been adjusted for in the including studies which used the multivariable analyses. (DOCX)
