# Supplementary material for: Prognostic value of FGFR1 expression and amplification in patients with HNSCC: A systematic review and meta-analysis
Source: PLoS One. 2021 May 14;16(5):e0251202. doi: 10.1371/journal.pone.0251202 (PMC8121309; doi:10.1371/journal.pone.0251202)
Supplement: S1 Table — (DOCX) [file pone.0251202.s002.docx]

| Author | Year | Selection | Comparability | Outcome | **NOS score** |
| --- | --- | --- | --- | --- | --- |
| Dubot [18] | 2018 | 3 | 2 | 2 | 7 |
| Koole 1[17] | 2016 | 4 | 2 | 2 | 8 |
| Monico[27] | 2018 | 3 | 1 | 2 | 6 |
| Young[33] | 2013 | 3 | 1 | 1 | 5 |
| Koole 2[32] | 2016 | 3 | 1 | 2 | 6 |
| Mariz[28] | 2019 | 3 | 1 | 2 | 6 |
| Starska[29] | 2018 | 3 | 2 | 1 | 6 |
| Kim[34] | 2020 | 3 | 2 | 2 | 7 |
| Goke[30] | 2013 | 3 | 2 | 1 | 6 |
