# Supplementary material for: Prognostic value of FGFR1 expression and amplification in patients with HNSCC: A systematic review and meta-analysis
Source: PLoS One. 2021 May 14;16(5):e0251202. doi: 10.1371/journal.pone.0251202 (PMC8121309; doi:10.1371/journal.pone.0251202)
Supplement: S2 Table — (DOCX) [file pone.0251202.s003.docx]

| **Author** | **Year** | **Cox proportional hazards mode** | **Covariates** |
| --- | --- | --- | --- |
| Dubot[25] | 2018 | Multivariate | HPV,tobacco,alcohol,UICC,TP53,CDKN2A,CCND1,genomic alterations |
| Koole1[28] | 2016 | Multivariate | alcohol，head and neck site,clinical T-classification,tumor stage,growth pattern,extra nodal growth,vaso-invasion |
| Monico[21] | 2018 | Multivariate | race,gender,T stage,N stage |
| Koole2[27] | 2016 | Multivariate | FGFR2,FGFR3,FGFR4,FGFR1-2,FGFR1-4 |
| Koole3[27] | 2016 | Multivariate | FGFR2,FGFR3,FGFR4,FGFR1-2,FGFR1-4 |
| Mariz[22] | 2019 | Multivariate | gender,age,smoking,alcohol,differentiation,tumour size,lymph node metastasis,distant metastasis,stage,vascular invasion,neural invasion,FGF-2 expression |
| Starska[23] | 2018 | Multivariate | FGFR3，p-PI3K，AKT，gender，smoking，alcohol，tumor size，differentiation，mode of invasion，depth of invasion，TFG score，SLUG |
